# Improvement of vertigo symptoms after 2 months of Vertigoheel treatment: a case series in patients with bilateral vestibulopathy and functional dizziness

**DOI:** 10.3389/fneur.2023.1264884

**Published:** 2023-10-05

**Authors:** Dilyana Ganeva, Rolf Tiemann, Stephan Duller, Michael Strupp

**Affiliations:** ^1^Department of Neurology, German Center for Vertigo and Balance Disorders, LMU University Hospital, LMU Munich, Munich, Germany; ^2^AMS Advanced Medical Services GmbH, Mannheim, Germany; ^3^Consultant, Graz, Austria

**Keywords:** vertigo, bilateral vestibulopathy, functional dizziness, real world evidence, non-interventional study, multicomponent medication

## Abstract

**Background:**

Dizziness is a common leading symptom in bilateral vestibulopathy (BVP) and functional dizziness (FD), with significant negative effects on functional ability and quality of life. Vertigoheel is a widely used non-prescription drug for the treatment of vertigo. In order to generate systematic data for Vertigoheel in BVP and FD, we conducted a non-interventional study assessing vertigo symptoms.

**Methods:**

This study was conducted as an open-label, prospective, monocentric, non-interventional case series (ClinicalTrials.gov identifier NCT05897853). Patients with BVP and FD received Vertigoheel according to market approval for an observational period of 2 months. Change from baseline after 2 months was assessed for the following endpoints: Dizziness Handicap Inventory (DHI) as the primary endpoint, quality of life (QoL) by EQ-5D-5L, and body sway by static posturography. Patients with FD were additionally assessed for depression and anxiety by PHQ-9 and GAD-7 questionnaires. Patients with BVP were assessed for vestibular function by video head impulse testing and caloric testing. Adverse events and other safety-related observations were evaluated.

**Results:**

Of 41 patients with FD and 13 with BVP, two with FD and none with BVP dropped out before the follow-up visit. Both patient groups showed significantly improved disability caused by dizziness after 2 months: In BVP, the DHI decreased on average by 13.2 points from 45.4 to 32.2 (*p* < 0.001). In FD, the DHI decreased on average by 12.0 points from 46.5 to 34.5 (*p* < 0.001). In patients with FD, significant improvements were also observed for the secondary endpoints QoL, anxiety, and depression. No significant change was observed for posturography readouts. In patients with BVP, there were no statistically significant improvements for the secondary endpoints QoL, posturography, or vestibular function within the observation period. The study found no evidence of a safety risk.

**Conclusion:**

The study provides evidence for Vertigoheel’s clinical safety and limited evidence – because of the non-interventional design – for its effectiveness in BVP and FD that are considered disease entities with high medical need for new treatment options. The results may serve as the basis for randomized placebo-controlled trials.

## 1. Introduction

Bilateral vestibulopathy (BVP) and functional dizziness (FD) are considered relevant disease entities with dizziness as a common leading symptom. Clinical epidemiologic data from the national neurological Special Vertigo Outpatient Clinic in Munich, Germany based on 37,328 patients between 1998 and 2020 showed relative frequencies for FD and BVP of 17.3 and 6.6%, respectively (1). BVP is characterized by chronic dizziness, unsteadiness, and oscillopsia due to bilateral impairment of the peripheral vestibular system (2, 3). FD is a more general term for somatoform or psychogenic dizziness as well as persistent postural-perceptual dizziness (4, 5). Dizziness has significant negative effects on functional ability and quality of life (6) and its prevalence reaches 30% beyond 60 years of age and 50% beyond 85 years of age (7). Regardless of the exact cause of dizziness, it is important to reduce the frequency, intensity, and duration of vertigo attacks with an effective medication that has minimal side effects. Vertigoheel is a natural non-prescription medicinal product containing four active ingredients for the treatment of vertigo and was approved by the German regulatory authority as a treatment for vertigo of various origins. According to recent research performed with rat models, Vertigoheel enhanced central vestibular compensation after unilateral peripheral vestibulopathy (8). Vertigoheel’s mode of action is not fully understood and under evaluation. One study proposed a vasodilating process, accompanied by an activation of the cyclic nucleotide signaling pathways (9). Another study, combining data from four other clinical studies, also supports the thesis that Vertigoheel is an effective and well-tolerated drug, in comparison with other therapies such as betahistine, *Ginkgo biloba* extract, and dimenhydrinate (10, 11).

To date, no systematic data for Vertigoheel on patient-reported outcomes (PRO) including quality of life (QoL) and objective measurements in BVP and FD were available. The objective of this study was to gain insight into the effects of Vertigoheel on patients suffering from BVP and FD in real life. PRO, including QoL and objective measurements were assessed during clinical practice and chosen as primary and secondary objectives, respectively, to evaluate symptoms and QoL of patients in Germany treated with Vertigoheel.

## 2. Materials and methods

### 2.1. Study design

This study was conducted as an open-label, prospective, monocentric, non-interventional study (case series) and was registered at ClinicalTrials.gov under NCT05897853. Vertigoheel was prescribed in the usual manner in accordance with the terms of the marketing authorization. The assignment of the patient to a particular therapeutic strategy was not defined by the observational plan but was the responsibility of the treating physician. No other types of interventions that would not otherwise be used were applied to the patients.

### 2.2. Patients

Patients with BVP and FD were recruited from the Department of Neurology at the Hospital of the LMU, Munich. The study population was composed of male and female patients, aged ≥18 years, diagnosed with BVP (3) or FD (5) according to the current diagnostic criteria of the Bárány Society. To be eligible, symptoms of moderate to severe intensity according to the DHI score between 30 and 90 must have been present for >3 months before inclusion. A cut-off of DHI <30 and > 90 was chosen to reduce any bottom and ceiling effects. Patients that had been taking Vertigoheel within the last 2 months were excluded. During the study, patients continued with their previous treatment in addition to Vertigoheel.

### 2.3. Outcome measures

The study participants underwent the examinations described below at baseline and after 2 ± 1 months of Vertigoheel treatment.

Disability resulting from dizziness and unsteadiness was quantified by the DHI (12), a 25-item questionnaire (range 0–100, the higher the worse). The scale has three sub-domains: physical (7 questions, maximal 28 points), functional (9 questions, maximal 36 points), and emotional questions (9 questions, maximal 36 points). The change from baseline (CFB) of the DHI total score was defined as the primary endpoint of the study. Results from the subdomains (physical/functional/emotional) were evaluated in an exploratory manner.

QoL was assessed by EQ-5D-5L (13). Results from the five dimensions (mobility, self-care, usual activities, pain/discomfort, and anxiety/depression) were tabulated and the numbers of patients shifting from one level to another were evaluated. An EQ-5D index value was built out of the data from the five dimensions based on the German Value Set for the EQ-5D-5L as described previously (14). In addition to the index, the VAS was used. For both the index and the VAS, CFB was evaluated as a secondary outcome measure.

Posturography was used to assess regulation of stance, with an artificial neural network analysis (15). A platform was used to measure body sway, which can exist in healthy subjects because of inherent physiological postural instability, and which is increased in vestibular disorders. Category three of the method for a “peripheral vestibular deficit” was evaluated for patients with BVP and category five for “phobic postural vertigo” (PPV) was evaluated for patients with FD. CFB was evaluated as a secondary outcome measure. Additionally, total sway path and root-mean-squared (RMS) sway variables were evaluated from the posturographic analysis. Patients with BVP were tested under conditions with eyes closed but no head reclination. Patients with FD were tested with eyes closed and head reclination of 30° up. A situation was chosen that was achievable for the majority of patients without assistance (16). Sway path and RMS sway were Ln transformed (natural logarithm of RMS sway) before statistical evaluation.

As FD is linked to psychiatric comorbidities, predominantly depressive or anxiety disorders (17), patients with FD were additionally assessed for depression and anxiety by using PHQ-9 (18, 19) and GAD-7 (20, 21) questionnaires. CFB of the total scores as well as shifts between categories to minimal, mild, moderate, and severe were evaluated.

In patients with BVP, the function of the vestibulo-ocular reflex (VOR) in the high frequency range for the horizontal semicircular canals was assessed by vHIT. The test is based on the clinical head impulse test, which is non-invasive, quick, and easy to perform, and does not generate unpleasant vertiginous or nauseating sensations for the patient. For the statistical analysis, values for left and right gain were averaged. CFB was then evaluated as a secondary outcome measure.

VOR in patients with BVP was also assessed by caloric testing, an established diagnostic tool for BVP (3). After a lesion of the eardrum had been ruled out, the patient’s head was positioned at an angle of 60° so that the horizontal semicircular canal was approximately vertical, thus ensuring maximum caloric excitability. Each external acoustic canal was then irrigated separately under standardized conditions with cool (30°C) and warm (44°C) water, while horizontal and vertical eye movements were recorded using electronystagmography. For the statistical analysis, absolute values from right warm/left warm/right cold/left cold were summed up. CFB was then evaluated as a secondary outcome measure.

Clinical safety was addressed by assessing AEs, physical examinations, and vital signs in a descriptive manner.

### 2.4. Statistics

Due to the non-interventional design, this study was set up for descriptive purposes, no *a priori* hypothesis was tested, and no comparisons were made with other products or treatments. All variables were analyzed in an exploratory manner and no formal statistical hypothesis testing was performed. Descriptive statistics were performed dependent on the type of data: For continuous and count variables, statistics include the number of observations, mean, standard deviation, median, minimum, maximum, and two-sided 95% confidence interval (CI). One-sample *t*-Tests were used to compare mean change from baseline values versus zero (no change). For categorical variables, the descriptive statistics include the count and percent of observations in each category along with its Clopper-Pearson two-sided 95% CI. Shift tables are given per baseline and 2-month value category as appropriate.

## 3. Results

### 3.1. Participants and baseline characteristics

A sample size of 40 FD and 20 patients with BVP was anticipated and a total of 62 patients completed the baseline visit. At baseline, the diagnostic criteria of the Bárány Society for BVP could not be confirmed for eight out of the 21 examined patients; these eight patients were therefore excluded from the analysis. In both conditions, we had more male than female patients, with 10 male vs. three female patients with BVP and 22 male vs. 19 female patients with FD. The age of the patients was 64.1 ± 11.2 in BVP and 64.4 ± 14.9 in FD. All but one patient with FD were defined as Caucasian. The number of patients, sex, medical history, concomitant medication, alcohol consumption, drug use, smoking behavior, ethnicity, age, weight, and height of all patients at baseline are given in Supplementary Table 2. Of the 41 patients with FD and 13 with BVP that attended the baseline visit, two patients with FD dropped out of the study and were lost to follow-up. A final set of 39 patients with FD and 13 with BVP completed the 2-month visit. The time period between baseline visits and the 2-month visits was on average 67.7 ± 20.4 days (median 63, min 32, max 174 days). Blood pressure (systolic and diastolic), heart rate, and weight at baseline and after 2 months are given in Supplementary Table 3. A total of 11 out of 54 patients in the study experienced 11 AEs. No serious AE occurred during the study and none of the AEs were categorized as related to the study drug. Four patients stopped the study drug administration, and one changed the dosage.

### 3.2. Dizziness handicap inventory (primary endpoint)

**Figure 1:**
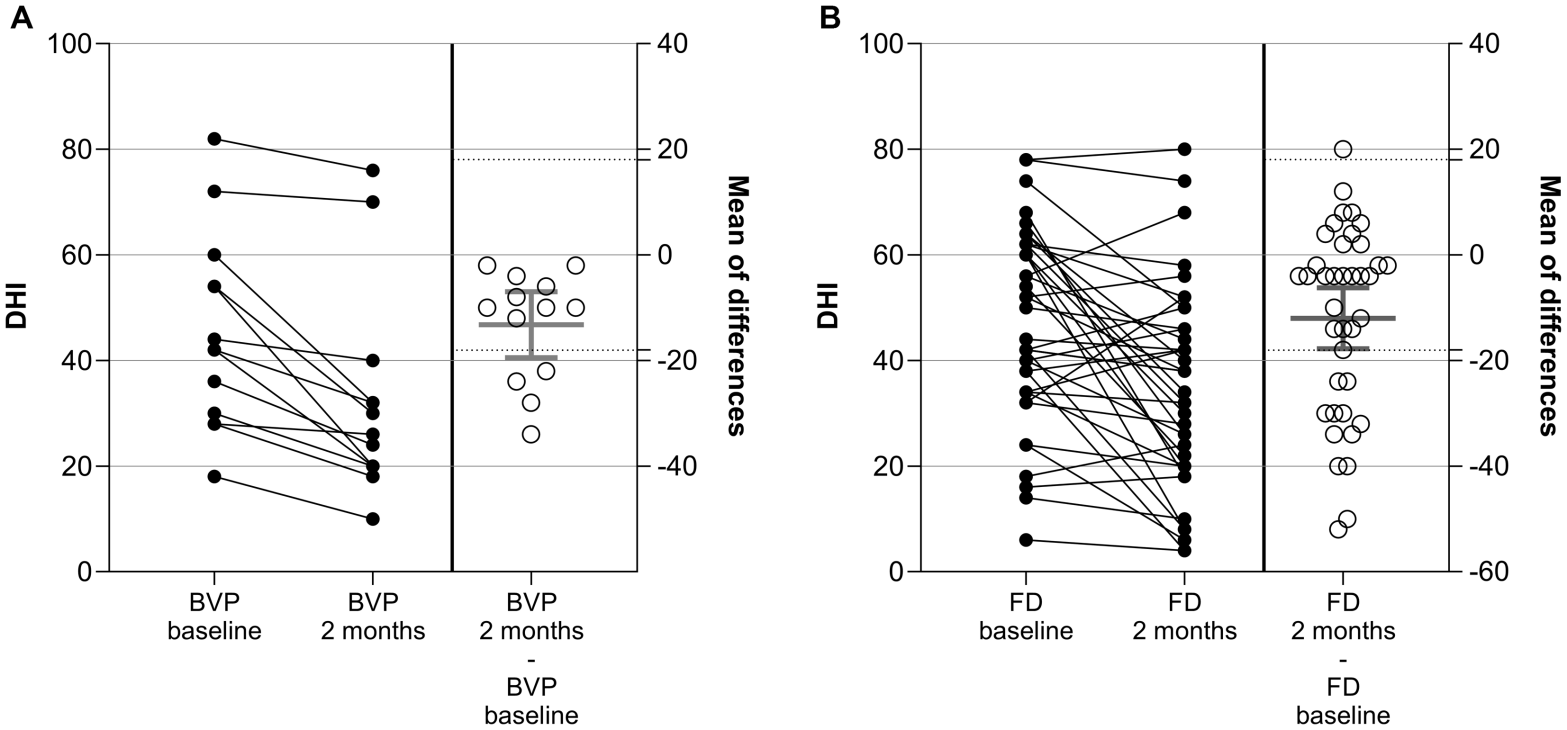
Dizziness handicap inventory (DHI) scores in patients with BVP **(A)** and FD **(B)** at baseline, after 2 ± 1 months, and as change from baseline (CFB; 2 ± 1 months – baseline). Each point represents data from one patient. For CFB, means with 95%CI are given. Dotted lines represent the minimally clinically important difference (MCID) of 18 points.

At baseline and after 2 months, disability resulting from dizziness and unsteadiness was evaluated by using DHI total scores. The CFB of the DHI score was defined as the primary endpoint of the study. Results are given in Figure 1 and Supplementary Table 4.

In patients with BVP, the mean DHI score declined on average by 13.2 points from 45.4 at baseline to 32.2 after 2 months (Figure 1A). The decrease was considered statistically significant in a one-sample *t*-Test (*p* < 0.001). A reduction (improvement) of the DHI score by ≥18 points, which is defined as the minimally clinically important difference (MCID) (12), was observed for four out of the 13 (31%) patients with BVP. None of the patients with BVP showed an increase (deterioration) of ≥ 18 points in the DHI score.

In patients with FD, the mean DHI score declined on average by 12.0 points from 46.5 at baseline to 34.5 after 2 months (Figure 1B). The decrease was considered statistically significant in a one-sample *t*-Test versus no change (*p* < 0.001). A reduction (improvement) of the DHI score by ≥18 points was observed for 13 out of the 39 (33%) patients with FD. A single patient with FD experienced an increase of ≥ 18 points in the DHI score (+20 points from 32 to 52).

The reduction of DHI score was observed in all three sub-domains, physical, emotional, and functional, at comparable effect sizes. Results for the subdomains are shown in Supplementary Table 5 and Supplementary Figure 1.

### 3.3. Quality of life (QoL) by EQ-5D-5L

**Figure 2:**
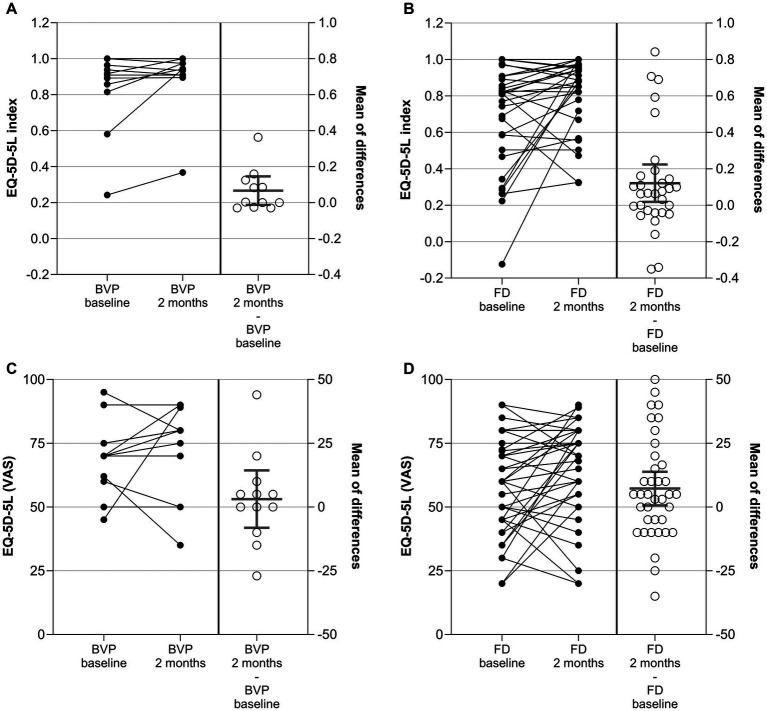
EQ-5D-5L index **(A,B)** and EQ-5D-5L VAS **(C,D)** in patients with BVP **(A,C)** and FD **(B,D)** at baseline, after 2 ± 1 months, and as change from baseline (CFB; 2 ± 1 months – baseline). Each point represents data from one patient. For the CFB, means with 95%CI are given.

The EQ-5D-5L was administered at the baseline and at the 2-month visit. An EQ-5D index value was built out of the data from the five dimensions (14). Additionally, the VAS of the EQ-5D-5L was used. For both variables, index and VAS, CFB were evaluated as secondary outcome measures. Results are given in Figure 2 and Supplementary Table 6.

In patients with BVP, the mean EQ-5D-5L index increased (improved) on average by 0.067 points from 0.829 at baseline to 0.895 after 2 months. The difference did not reach the level of significance in one-sample *t*-Test (*p* = 0.09 versus no change). The VAS score did not change significantly between baseline and the 2-month visits for the patients with BVP either.

In patients with FD, the mean EQ-5D-5L index increased (improved) on average by 0.121 points from 0.693 at baseline to 0.814 after 2 months (*p* = 0.02). In line with this, the VAS score improved by 7.2 points from 57.8 to 66.1 points (*p* = 0.03).

The distribution of EQ-5D-5L dimension responses per dimension and level at baseline and after 2 months is given in Supplementary Table 7. In the BVP cohort, six (of 12) patients switched to lower levels (improvement) in the mobility dimension (D1), whereas four stayed at the same level and two switched to a higher level. The improvements for the other domains in the BVP cohort were less prominent. In the FD cohort, a high number of patients switching to a lower level was observed for the dimensions mobility (16 out of 33), usual activities (17 out of 33), and anxiety/depression (17 out of 33). A shift table is given for dichotomized (no problem vs. any problem) EQ-5D-5L data in Supplementary Table 8. The dichotomized levels at baseline and after 2 months were compared by McNemar’s test. A significant result was observed for usual activities in the FD cohort only (*p* = 0.04).

### 3.4. Posturography

Posturography was used to assess the regulation of stance and gait. Category three of the method for “*peripheral vestibular deficit*” was evaluated for patients with BVP, and category five for “*phobic postural vertigo*” (PPV) was evaluated for patients with FD. Measurements were performed at the baseline visit and at the 2-month visit. CFB was evaluated as a secondary outcome measure. No significant changes from baseline were observed for these variables (Supplementary Table 9 and Supplementary Figure 2). Additionally, total sway path and RMS sway variables were evaluated from the posturographic analysis. No significant changes from baseline were observed for these variables either (Supplementary Table 10 and Supplementary Figure 3).

### 3.5. Anxiety and depression in patients with FD

**Figure 3:**
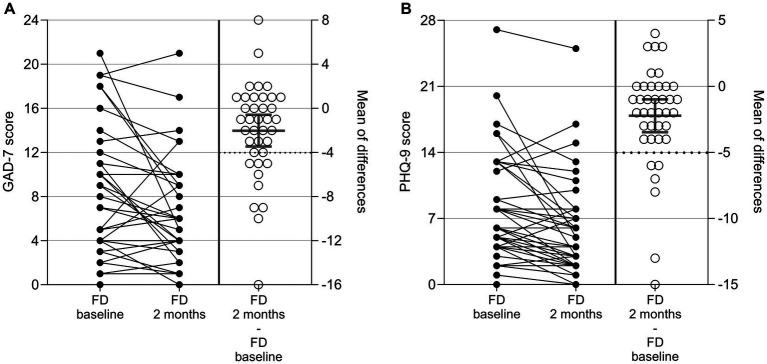
GAD-7 **(A)** and PHQ-9 **(B)** results in patients with FD at baseline, after 2 ± 1 months, and as change from baseline (CFB; 2 ± 1 months – baseline). Each point represents data from one patient. For the CFB, means with 95%CI are given.

Anxiety and depression in patients with FD were assessed by GAD-7 and PHQ-9 questionnaires at baseline and after 2 months. CFB was evaluated as a secondary outcome measure. Results are given in Figure 3 and Supplementary Table 11.

The GAD-7 total score declined on average by 2.0 points from 8.4 at baseline to 6.3 after 2 months. The decrease was considered statistically significant in a one-sample *t*-Test versus no change (*p* = 0.01). A reduction (improvement) by ≥4 points, which is defined as the MCID (22), was observed for 11 out of the 38 patients (29%) in this analysis. Two patients showed an increase of ≥ 4 points (deterioration) in the GAD-7 score. The number of patients in each of the four categories (minimal, mild, moderate, and severe anxiety) at baseline and after 2 months as well as the number shifting from one into the other are shown in Supplementary Table 12. Of the 38 patients in the analysis, 25 (66%) remained in the same category at baseline and after 2 months, 11 (29%) improved toward a lower anxiety category, and two patients (5%) shifted to a higher anxiety category.

The PHQ-9 score decreased on average by 2.2 points from 8.2 at baseline to 5.9 after 2 months. The decrease was considered statistically significant in a one-sample *t*-Test versus no change (*p* < 0.001). A reduction (improvement) by ≥5 points, which is defined as the MCID (19), was observed for six out of the 39 patients (15%) in this analysis. None of the patients showed an increase of ≥ 5 points in the PHQ-9 score. The number of patients in each of the four categories (minimal, mild, moderate, and severe anxiety) at baseline and after 2 months as well as the number shifting from one into the other are shown in Supplementary Table 13. Of the 39 patients in the analysis, 21 (54%) remained in the same category, 14 (36%) improved to a lower depressive category, and four patients (10%) shifted to a higher depressive category 2 months after baseline.

### 3.6. Vestibular function in patients with BVP

Patients with BVP were assessed for vestibular function by vHIT testing and caloric testing. No significant change from baseline was observed for the vHIT variable or for the caloric testing (Supplementary Table 14 and Supplementary Figure 4).

## 4. Discussion

Within an open-label, prospective, non-interventional case series, we followed 13 patients with BVP and 41 patients with FD receiving the non-prescription drug Vertigoheel over a period of 2 months. We could show that the disability resulting from dizziness and unsteadiness as measured by the DHI score as the primary outcome improved significantly within the 2 months of Vertigoheel treatment in both patient groups. In patients with BVP, the mean DHI score declined on average by 13.2 points from 45.4 at baseline to 32.24 after 2 months (*p* < 0.001). In patients with FD, the mean DHI score declined on average by 12.0 points from 46.5 to 34.5 (*p* < 0.001). In 31 and 33% of the patients with BVP and FD respectively, the improvement was ≥18 points, which is regarded as clinically meaningful.

Significant improvements were also observed in patients with FD for the secondary endpoints QoL, anxiety, and depression. No significant change was observed for posturography readouts. In patients with BVP, we could not show any statistically significant improvements for the secondary endpoints QoL, posturography, or vestibular function, within the observation period. The missing effect on QoL in patients with BVP might be attributable to the low sample size (*n* = 13) for the patient group.

During the 2-month observation of 54 patients, no SAE and 11 AEs occurred. None of the AEs were categorized as related to the study drug. Four patients stopped the study drug administration, and one changed the dosage. Thus, the study drug can be regarded as safe and well tolerated. The study showed no indication for a safety risk.

This case series gave some evidence that a 2-month treatment with Vertigoheel for patients with FD and BVP (both with the leading symptom of persisting dizziness) may improve the patients’ symptoms in real life. Due to the nature of non-interventional studies, the evidence for effectiveness from the current study might still be seen as insufficient to recommend treatment with Vertigoheel in daily practice. However, in BVP, Vertigoheel could be a complementary treatment to vestibular exercises, the established therapy (23). The same is true for the treatment of FD, although there is a lack of well-designed placebo-controlled trials for standard therapies (24–27).

In preclinical studies, it was shown that Vertigoheel improves central vestibular compensation (8). However, the mechanism of action in BVP and FD is so far largely unclear and should be examined in further studies.

Overall, the study provides evidence for the study drug’s clinical safety and limited evidence - because of the study design – for its effectiveness in patients with persisting dizziness due to FD or BVP. The results encourage further studies, including randomized controlled trials, to complement this real-life evidence.

### 4.1. Limitations

The design of this study is non-interventional with no control group. The study was performed at a single center in Germany.

## 5. Conclusion

Treatment with Vertigoheel over a period of 2 months may improve the symptoms of patients with FD and patients with BVP. However, the limitations of a non-interventional, observational study design must be considered. There were no relevant safety findings. The results of this NIS can be the basis for a prospective randomized placebo-controlled trial with the same dosage and treatment period.

## Data availability statement

The raw data supporting the conclusions of this article will be made available by the authors, without undue reservation.

## Ethics statement

The studies involving humans were approved by Ethics Committees of the Faculty of Medicine at Ludwig Maximilian University, Munich, Germany. The studies were conducted in accordance with the local legislation and institutional requirements. The participants provided their written informed consent to participate in this study.

## Author contributions

DG: Investigation, Writing – original draft, Writing – review & editing. RT: Formal analysis, Writing – review & editing. SD: Visualization, Writing – review & editing, Formal analysis. MS: Conceptualization, Supervision, Writing – review & editing.
