# Supplementary material for: Improvement of vertigo symptoms after 2 months of Vertigoheel treatment: a case series in patients with bilateral vestibulopathy and functional dizziness
Source: Front Neurol. 2023 Oct 5;14:1264884. doi: 10.3389/fneur.2023.1264884 (PMC10586313; doi:10.3389/fneur.2023.1264884)
Supplement: Supplementary file 1 [file Table_1.DOCX]

Supplementary Material

Improvement of vertigo symptoms after two months Vertigoheel treatment: A case series in patients with bilateral vestibulopathy and functional dizziness

Dilyana Ganeva, Rolf Tiemann, Stephan Duller, Michael Strupp*

*** Correspondence:** Corresponding Author: Michael.Strupp@med.uni-muenchen.de

# Supplementary Tables

Supplementary Table 1: Conditions for stance testing during posturography

|  | Eyes | Head inclination | Foam pad | Stance |
| --- | --- | --- | --- | --- |
| 1 | open | no | no | side by side 30° |
| 2 | closed | no | no | side by side 30° |
| 3 | open | 30° up | no | side by side 30° |
| 4 | closed | 30° up | no | side by side 30° |
| 5 | open | no | yes | side by side 30° |
| 6 | closed | no | yes | side by side 30° |
| 7 | open | 30° up | yes | side by side 30° |
| 8 | closed | 30° up | yes | side by side 30° |
| 9 | open | no | yes | tandem |
| 10 | closed | no | yes | tandem |

Supplementary Table 2: Number of patients, sex, medical history, concomitant medication, alcohol consumption, drug use, smoking behavior, ethnicity, age, weight, and height of all patients as well as of patients with bilateral vestibulopathy (BVP) and Functional dizziness (FD) at baseline. Data are given as counts and percent of observations or mean, standard deviation (SD), median, minimum (min), and maximum (max).

| **Demographics** |  | All | Bilateral vestibulopathy | Functional dizziness |
| --- | --- | --- | --- | --- |
| Number of patients | | 54 | 13 | 41 |
| Sex [n (%)] | female | 22 (40.7%) | 3 (23.1%) | 19 (46.3%) |
|  | male | 32 (59.3%) | 10 (76.9%) | 22 (53.7%) |
| Medical history [n (%)] | yes | 48 (88.9%) | 11 (84.6%) | 37 (90.2%) |
|  | no | 6 (11.1%) | 2 (15.4%) | 4 (9.8%) |
| Concomitant medication [n (%)] | yes | 44 (81.5%) | 10 (76.9%) | 34 (82.9%) |
|  | no | 10 (18.5%) | 3 (23.1%) | 7 (17.1%) |
| Alcohol consumption [n (%)] | never | 12 (22.2%) | 4 (30.8%) | 8 (19.5%) |
|  | occasional | 33 (61.1%) | 7 (53.8%) | 26 (63.4%) |
|  | regularly | 9 (16.7%) | 2 (15.4%) | 7 (17.1%) |
|  | no information | 0 (0%) | 0 (0%) | 0 (0%) |
| Drug use [n (%)] | never | 53 (98.1%) | 13 (100%) | 40 (97.6%) |
|  | occasional | 1 (1.9%) | 0 (0%) | 1 (2.4%) |
|  | regularly | 0 (0%) | 0 (0%) | 0 (0%) |
| Smoking [n (%)] | never | 43 (79.6%) | 11 (84.6%) | 32 (78%) |
|  | occasional | 2 (3.7%) | 0 (0%) | 2 (4.9%) |
|  | ex | 8 (14.8%) | 1 (7.7%) | 7 (17.1%) |
|  | regularly | 1 (1.9%) | 1 (7.7%) | 0 (0%) |
| Ethnicity [n (%)] | caucasian | 53 (98.1%) | 13 (100%) | 40 (97.6%) |
|  | other* | 1 (1.9%) | 0 (0%) | 1 (2.4%) |
| Age [years] | mean ± SD | 64.3 ± 14 | 64.1 ± 11.2 | 64.4 ± 14.9 |
|  | median (min - max) | 67 (29 - 87) | 68 (44 - 78) | 67 (29 - 87) |
| Weight [kg] | mean ± SD | 76.6 ± 16 | 86 ± 21.6 | 73.7 ± 12.8 |
|  | median (min - max) | 74 (51 - 123) | 87 (54 - 123) | 72 (51 - 102) |
| Height [cm] | mean ± SD | 175.4 ± 10.6 | 179.5 ± 11.1 | 174 ± 10.3 |
|  | median (min - max) | 177 (155 - 198) | 181 (161 - 198) | 174 (155 - 191) |

*one patient self defined as “Arabic”

Supplementary Table 3: Blood pressure (systolic and diastolic), heart rate, and weight of patients with BVP and FD at baseline, after 2±1 months, and as change from baseline (CFB). Data are given as number of patients with available data for both time points and each readout (n), mean, standard deviation (SD), upper and lower 95% confidence interval (CI) of the mean, median, minimum (min), and maximum (max).

| **Vital signs** | | Bilateral vestibulopathy | | | Functional dizziness | | |
| --- | --- | --- | --- | --- | --- | --- | --- |
|  |  | baseline | 2 months | CFB | baseline | 2 months | CFB |
| Systolic blood pressure [mmHg] | n | 13 | 13 | 13 | 28 | 28 | 28 |
|  | **mean** | **140.2** | **138.8** | **-1.3** | **142.3** | **137.9** | **-4.4** |
|  | SD | 21.2 | 21.2 | 9.7 | 21.2 | 18.5 | 13.3 |
|  | lower 95%CI | 127.3 | 126.0 | -7.2 | 134.1 | 130.7 | -9.5 |
|  | upper 95%CI | 153.0 | 151.7 | 4.6 | 150.5 | 145.1 | 0.8 |
|  | median | 138 | 136 | 0 | 143 | 136 | -5 |
|  | min | 111 | 113 | -16 | 107 | 110 | -40 |
|  | max | 175 | 180 | 11 | 195 | 181 | 14 |
| Diastolic blood pressure [mmHg] | n | 13 | 13 | 13 | 28 | 28 | 28 |
|  | **mean** | **90.7** | **90.6** | **-0.1** | **93.1** | **88.5** | **-4.7** |
|  | SD | 9.9 | 10.7 | 7.9 | 10.2 | 10.4 | 7.3 |
|  | lower 95%CI | 84.7 | 84.1 | -4.9 | 89.2 | 84.4 | -7.5 |
|  | upper 95%CI | 96.7 | 97.1 | 4.7 | 97.1 | 92.5 | -1.8 |
|  | median | 90 | 91 | 1 | 91.5 | 87.5 | -4 |
|  | min | 75 | 67 | -14 | 76 | 72 | -23 |
|  | max | 103 | 107 | 14 | 116 | 113 | 7 |
| Heart rate [bpm] | n | 13 | 13 | 13 | 27 | 27 | 27 |
|  | **mean** | **67.6** | **68.1** | **0.5** | **71.0** | **72.8** | **1.7** |
|  | SD | 8.4 | 10.6 | 5.5 | 11.5 | 11.5 | 11.2 |
|  | lower 95%CI | 62.6 | 61.7 | -2.8 | 66.5 | 68.2 | -2.7 |
|  | upper 95%CI | 72.7 | 74.5 | 3.8 | 75.6 | 77.3 | 6.2 |
|  | median | 70 | 68 | -1 | 70 | 71 | -1 |
|  | min | 50 | 47 | -7 | 53 | 57 | -22 |
|  | max | 81 | 93 | 12 | 97 | 96 | 28 |
| Weight [kg] | n | 12 | 12 | 12 | 30 | 30 | 30 |
|  | **mean** | **86.0** | **85.5** | **-0.5** | **75.0** | **75.2** | **0.2** |
|  | SD | 21.6 | 21.6 | 1.2 | 13.8 | 13.7 | 1.8 |
|  | lower 95%CI | 72.3 | 71.8 | -1.3 | 69.8 | 70.1 | -0.4 |
|  | upper 95%CI | 99.7 | 99.2 | 0.3 | 80.2 | 80.4 | 0.9 |
|  | median | 87 | 87.5 | 0 | 74.5 | 74.5 | 0 |
|  | min | 54 | 54 | -4 | 51 | 51 | -3 |
|  | max | 123 | 123 | 1 | 102 | 102 | 5 |

Supplementary Table 4: Dizziness handicap inventory (DHI) scores in patients with BVP and FD at baseline, after 2±1 months, and as change from baseline (CFB). Data are given as number of patients with available data for both collection time points (n), mean, standard deviation (SD), upper and lower 95% confidence interval (CI) of the mean, median, minimum (min), maximum (max), One sample t-Test p value for CFB vs no change as well as number (n), proportion and 95%CI of the proportion of patients with DHI reduction of ≥18 points.

| **DHI score** | Bilateral vestibulopathy | | | Functional dizziness | | |
| --- | --- | --- | --- | --- | --- | --- |
|  | baseline | 2 months | CFB | baseline | 2 months | CFB |
| n | 13 | 13 | 13 | 39 | 39 | 39 |
| **mean** | **45.4** | **32.2** | **-13.2** | **46.5** | **34.5** | **-12.0** |
| SD | 18.5 | 19.7 | 10.4 | 18.1 | 19.0 | 17.8 |
| lower 95%CI | 34.2 | 20.2 | -19.5 | 40.7 | 28.3 | -17.8 |
| upper 95%CI | 56.6 | 44.1 | -7.0 | 52.4 | 40.7 | -6.2 |
| median | 42 | 26 | -10 | 44 | 34 | -4 |
| min | 18 | 10 | -34 | 6 | 4 | -52 |
| max | 82 | 76 | -2 | 78 | 80 | 20 |
| One sample T-Test p value for CFB vs no change | - | - | <0.001 | - | - | <0.001 |
| n patients with reduction ≥18 | - | - | 4 | - | - | 13 |
| proportion of patients with reduction ≥18 | - | - | 31% | **-** | **-** | 33% |
| upper Clopper-Pearson 95%CI | - | - | 9% | - | - | 19% |
| lower Clopper-Pearson 95%CI | - | - | 61% | - | - | 50% |

Supplementary Table 5: Dizziness handicap inventory (DHI) scores for physical, emotional, and functional sub-domains in patients with BVP and FD at baseline, after 2±1 months, and as change from baseline (CFB). Data are given as number of patients with available data for both collection time points (n), mean, standard deviation (SD), upper and lower 95% confidence interval (CI) of the mean, median, minimum (min), maximum (max) as well as One sample t-Test p value for CFB vs no change.

| **DHI score subdomains** | | Bilateral vestibulopathy | | | Functional dizziness | | |
| --- | --- | --- | --- | --- | --- | --- | --- |
|  |  | baseline | 2 months | CFB | baseline | 2 months | CFB |
| subdomain physical | n | 13 | 13 | 13 | 39 | 39 | 39 |
|  | **mean** | **14.6** | **10.3** | **-4.3** | **13.4** | **10.7** | **-2.7** |
|  | SD | 7.2 | 7.2 | 4.2 | 6.2 | 7.0 | 5.5 |
|  | lower 95%CI | 10.2 | 6.0 | -6.9 | 11.4 | 8.5 | -4.5 |
|  | upper 95%CI | 19.0 | 14.7 | -1.8 | 15.4 | 13.0 | -0.9 |
|  | median | 16 | 8 | -4 | 14 | 8 | -2 |
|  | min | 4 | 2 | -12 | 0 | 0 | -16 |
|  | max | 28 | 28 | 2 | 26 | 24 | 12 |
|  | One sample T-Test p value for CFB vs no change | - | - | 0.0032 | - | - | 0.0039 |
| subdomain emotional | n | 13 | 13 | 13 | 39 | 39 | 39 |
|  | **mean** | **13.2** | **10.5** | **-2.8** | **15.7** | **11.3** | **-4.5** |
|  | SD | 6.1 | 6.3 | 4.2 | 8.3 | 8.0 | 7.3 |
|  | lower 95%CI | 9.5 | 6.6 | -5.3 | 13.0 | 8.7 | -6.8 |
|  | upper 95%CI | 16.9 | 14.3 | -0.2 | 18.5 | 13.9 | -2.1 |
|  | median | 12 | 8 | -4 | 16 | 10 | -4 |
|  | min | 2 | 4 | -8 | 4 | 0 | -22 |
|  | max | 22 | 26 | 4 | 36 | 36 | 8 |
|  | One sample T-Test p value for CFB vs no change | - | - | 0.0352 | - | - | <0.001 |
| subdomain functional | n | 13 | 13 | 13 | 39 | 39 | 39 |
|  | **mean** | **17.5** | **11.4** | **-6.2** | **17.3** | **12.5** | **-4.8** |
|  | SD | 6.8 | 7.5 | 5.3 | 7.4 | 7.9 | 7.7 |
|  | lower 95%CI | 13.4 | 6.9 | -9.4 | 14.9 | 10.0 | -7.3 |
|  | upper 95%CI | 21.7 | 15.9 | -2.9 | 19.7 | 15.1 | -2.3 |
|  | median | 16 | 12 | -6 | 16 | 12 | -4 |
|  | min | 8 | 2 | -14 | 0 | 0 | -24 |
|  | max | 32 | 28 | 6 | 30 | 30 | 8 |
|  | One sample T-Test p value for CFB vs no change | - | - | 0.0013 | - | - | <0.001 |

Supplementary Table 6: EQ-5D-5L index and VAS in patients with BVP and FD at baseline, after 2±1 months, and as change from baseline (CFB). Data are given as number of patients with available data for both collection time points (n), mean, standard deviation (SD), upper and lower 95% confidence interval (CI) of the mean, median, minimum (min), maximum (max), One sample t-Test p value for CFB vs no change.

| **EQ-5D-5L** | | Bilateral vestibulopathy | | | Functional dizziness | | |
| --- | --- | --- | --- | --- | --- | --- | --- |
|  |  | baseline | 2 months | CFB | baseline | 2 months | CFB |
| index | n | 11 | 11 | 11 | 31 | 31 | 31 |
|  | **mean** | **0.829** | **0.895** | **0.067** | **0.693** | **0.814** | **0.121** |
|  | SD | 0.227 | 0.179 | 0.119 | 0.283 | 0.199 | 0.280 |
|  | lower 95%CI | 0.676 | 0.775 | -0.013 | 0.589 | 0.741 | 0.018 |
|  | upper 95%CI | 0.981 | 1.015 | 0.146 | 0.797 | 0.887 | 0.223 |
|  | median | 0.909 | 0.943 | 0.003 | 0.813 | 0.879 | 0.066 |
|  | min | 0.242 | 0.367 | -0.030 | -0.124 | 0.323 | -0.351 |
|  | max | 1.000 | 1.000 | 0.363 | 1.000 | 1.000 | 0.842 |
|  | One sample T-Test p value for CFB vs no change | - | - | 0.0923 | - | - | 0.0224 |
| VAS | n | 12 | 12 | 12 | 37 | 37 | 37 |
|  | **mean** | **69.3** | **72.4** | **3.1** | **57.8** | **65.1** | **7.2** |
|  | SD | 14.3 | 17.9 | 17.7 | 19.0 | 18.8 | 19.8 |
|  | lower 95%CI | 60.2 | 61.0 | -8.2 | 51.5 | 58.8 | 0.6 |
|  | upper 95%CI | 78.4 | 83.8 | 14.3 | 64.2 | 71.3 | 13.8 |
|  | median | 70 | 80 | 2.5 | 60 | 70 | 5 |
|  | min | 45 | 35 | -27 | 20 | 20 | -35 |
|  | max | 95 | 90 | 44 | 90 | 90 | 50 |
|  | One sample T-Test p value for CFB vs no change | - | - | 0.5587 | - | - | 0.0328 |

Supplementary Table 7: Distribution of EQ-5D-5L dimension responses at baseline and after 2±1 months in patients with BVP and FD. Data are given as counts and percent per dimension and level. Additionally number and percent of patients are given that stayed at the same level or improved or worsened by 1 level or more after 2±1 months.

| **EQ-5D-5L** | | Bilateral vestibulopathy | | Functional dizziness | |
| --- | --- | --- | --- | --- | --- |
|  |  | BL | 2mo | BL | 2mo |
| Mobility (D1) | **No problems (1)** | **4 (33%)** | **4 (33%)** | **7 (21%)** | **12 (36%)** |
|  | **Any problems (2-5)** | **8 (67%)** | **8 (67%)** | **26 (79%)** | **21 (64%)** |
|  | Slight problems (2) | 3 (25%) | 6 (50%) | 6 (18%) | 10 (30%) |
|  | Moderate problems (3) | 3 (25%) | 2 (17%) | 15 (45%) | 7 (21%) |
|  | Severe problems (4) | 2 (17%) | 0 (0%) | 5 (15%) | 4 (12%) |
|  | Unable to walk about (5) | 0 (0%) | 0 (0%) | 0 (0%) | 0 (0%) |
|  | stayed at same level | 4 (33%) | | 14 (42%) | |
|  | improved by ≥ 1 level | 6 (50%) | | 16 (48%) | |
|  | worsened by ≥ 1 level | 2 (17%) | | 3 (9%) | |
| Self-care (D2) | **No problems (1)** | **9 (75%)** | **11 (92%)** | **28 (85%)** | **28 (85%)** |
|  | **Any problems (2-5)** | **3 (25%)** | **1 (8%)** | **5 (15%)** | **5 (15%)** |
|  | Slight problems (2) | 2 (17%) | 0 (0%) | 3 (9%) | 2 (6%) |
|  | Moderate problems (3) | 1 (8%) | 1 (8%) | 2 (6%) | 3 (9%) |
|  | Severe problems (4) | 0 (0%) | 0 (0%) | 0 (0%) | 0 (0%) |
|  | Unable to wash or dress (5) | 0 (0%) | 0 (0%) | 0 (0%) | 0 (0%) |
|  | stayed at same level | 10 (83%) | | 29 (88%) | |
|  | improved by ≥ 1 level | 2 (17%) | | 2 (6%) | |
|  | worsened by ≥ 1 level | 0 (0%) | | 2 (6%) | |
| Usual activities (D3) | **No problems (1)** | **3 (25%)** | **6 (50%)** | **7 (21%)** | **15 (45%)** |
|  | **Any problems (2-5)** | **8 (67%)** | **5 (42%)** | **25 (76%)** | **17 (52%)** |
|  | Slight problems (2) | 6 (50%) | 2 (17%) | 9 (27%) | 8 (24%) |
|  | Moderate problems (3) | 1 (8%) | 3 (25%) | 6 (18%) | 6 (18%) |
|  | Severe problems (4) | 1 (8%) | 0 (0%) | 9 (27%) | 3 (9%) |
|  | Unable to do usual activities (5) | 0 (0%) | 0 (0%) | 1 (3%) | 0 (0%) |
|  | stayed at same level | 6 (55%) | | 12 (38%) | |
|  | improved by ≥ 1 level | 4 (36%) | | 17 (53%) | |
|  | worsened by ≥ 1 level | 1 (9%) | | 3 (9%) | |
| Pain/discomfort (D4) | **No problems (1)** | **8 (67%)** | **9 (75%)** | **16 (48%)** | **12 (36%)** |
|  | **Any problems (2-5)** | **4 (33%)** | **3 (25%)** | **16 (48%)** | **20 (61%)** |
|  | Slight problems (2) | 2 (17%) | 1 (8%) | 3 (9%) | 12 (36%) |
|  | Moderate problems (3) | 0 (0%) | 0 (0%) | 7 (21%) | 6 (18%) |
|  | Severe problems (4) | 2 (17%) | 2 (17%) | 5 (15%) | 2 (6%) |
|  | Extreme pain/discomfort (5) | 0 (0%) | 0 (0%) | 1 (3%) | 0 (0%) |
|  | stayed at same level | 9 (75%) | | 10 (31%) | |
|  | improved by ≥ 1 level | 2 (17%) | | 12 (38%) | |
|  | worsened by ≥ 1 level | 1 (8%) | | 10 (31%) | |
| Anxiety/depression (D5) | **No problems (1)** | **8 (67%)** | **7 (58%)** | **6 (18%)** | **13 (39%)** |
|  | **Any problems (2-5)** | **4 (33%)** | **5 (42%)** | **27 (82%)** | **20 (61%)** |
|  | Slight problems (2) | 3 (25%) | 4 (33%) | 10 (30%) | 14 (42%) |
|  | Moderate problems (3) | 0 (0%) | 1 (8%) | 10 (30%) | 3 (9%) |
|  | Severe problems (4) | 1 (8%) | 0 (0%) | 5 (15%) | 2 (6%) |
|  | Extremely anxious/depressed (5) | 0 (0%) | 0 (0%) | 2 (6%) | 1 (3%) |
|  | stayed at same level | 7 (58%) | | 13 (39%) | |
|  | improved by ≥ 1 level | 2 (17%) | | 17 (52%) | |
|  | worsened by ≥ 1 level | 3 (25%) | | 3 (9%) | |

Supplementary Table 8: Dichotomized (no problem vs any problem) EQ-5D-5L shift table: Number of patients (n) and proportion (%) shifted between no problem and any problem after 2±1 months from baseline for the 5 dimensions. McNemar's test p value for the contingency tables are given.

| **EQ-5D-5L (dichotomized) Shift tables** | | Bilateral vestibulopathy | | | Functional dizziness | | |
| --- | --- | --- | --- | --- | --- | --- | --- |
|  |  | 2mo - No problems (1) | 2 mo - Any problems (2-5) | McNemar's test p value | 2mo - No problems (1) | 2 mo - Any problems (2-5) | McNemar's test p value |
| Mobility  D1 | BL - No problems (1) | 2 (17%) | 2 (17%) | 0.6171 | 5 (15%) | 2 (6%) | 0.1824 |
|  | BL - Any problems (2-5) | 2 (17%) | 6 (50%) |  | 7 (21%) | 19 (58%) |  |
| Self-care  D2 | BL - No problems (1) | 9 (75%) | 0 (0%) | 0.4795 | 27 (82%) | 1 (3%) | 0.4795 |
|  | BL - Any problems (2-5) | 2 (17%) | 1 (8%) |  | 1 (3%) | 4 (12%) |  |
| Usual activities D3 | BL - No problems (1) | 3 (27%) | 0 (0%) | 0.2482 | 5 (16%) | 2 (6%) | **0.0433** |
|  | BL - Any problems (2-5) | 3 (27%) | 5 (45%) |  | 10 (31%) | 15 (47%) |  |
| Pain/discomfort D4 | BL - No problems (1) | 7 (58%) | 1 (8%) | 1.000 | 7 (22%) | 9 (28%) | 0.4227 |
|  | BL - Any problems (2-5) | 2 (17%) | 2 (17%) |  | 5 (16%) | 11 (34%) |  |
| Anxiety/depres-sion D5 | BL - No problems (1) | 6 (50%) | 2 (17%) | 1.000 | 4 (12%) | 2 (6%) | 0.0704 |
|  | BL - Any problems (2-5) | 1 (8%) | 3 (25%) |  | 9 (27%) | 18 (55%) |  |

Supplementary Table 9: Posturography measured in patients with BVP and FD at baseline, after 2±1 months, and as change from baseline (CFB). Data are given as number of patients with available data for both collection time points (n), mean, standard deviation (SD), upper and lower 95% confidence interval (CI) of the mean, median, minimum (min), maximum (max), One sample t-Test p value for CFB vs no change.

| **Posturography** | | Bilateral vestibulopathy (cat3 - vestibular deficit) | | | Functional dizziness (cat5 - PPV) | | |
| --- | --- | --- | --- | --- | --- | --- | --- |
|  |  | baseline | 2 months | CFB | baseline | 2 months | CFB |
| score | n | 13 | 13 | 13 | 28 | 28 | 28 |
|  | **mean** | **18.2** | **18.3** | **0.2** | **24.9** | **27.6** | **2.8** |
|  | SD | 10.8 | 9.1 | 8.3 | 14.4 | 17.8 | 16.9 |
|  | lower 95%CI | 11.6 | 12.8 | -4.9 | 19.3 | 20.7 | -3.8 |
|  | upper 95%CI | 24.7 | 23.8 | 5.2 | 30.5 | 34.6 | 9.3 |
|  | median | 19 | 16 | 1 | 21.5 | 25.5 | 0 |
|  | min | 4 | 8 | -12 | 6 | 5 | -31 |
|  | max | 38 | 39 | 17 | 55 | 57 | 42 |
|  | One sample T-Test p value for CFB vs no change | - | - | 0.9480 | - | - | 0.3961 |

Supplementary Table 10: Swaypath and RMS sway variables from posturographic analysis measured in patients with BVP and FD at baseline, after 2±1 months, and as change from baseline (CFB). Data are given as number of patients with available data for both collection time points (n), mean, standard deviation (SD), upper and lower 95% confidence interval (CI) of the mean, median, minimum (min), maximum (max), One sample t-Test p value for CFB vs no change.

| **Posturography** | | **Bilateral vestibulopathy** Condition 2 Eyes closed / no head reclination | | | **Functional dizziness** Condition 3 Eyes open / head reclination 30° up | | |
| --- | --- | --- | --- | --- | --- | --- | --- |
|  |  | baseline | 2 months | CFB | baseline | 2 months | CFB |
| Ln total sway path [m/min] | n | 13 | 13 | 13 | 27 | 27 | 27 |
|  | **mean** | **0.974** | **0.937** | **-0.037** | **0.253** | **0.233** | **-0.021** |
|  | SD | 0.688 | 0.695 | 0.433 | 0.337 | 0.382 | 0.352 |
|  | lower 95%CI | 0.559 | 0.517 | -0.299 | 0.120 | 0.081 | -0.160 |
|  | upper 95%CI | 1.390 | 1.357 | 0.224 | 0.387 | 0.384 | 0.119 |
|  | median | 0.811 | 0.942 | -0.074 | 0.201 | 0.160 | 0.040 |
|  | min | 0.129 | 0.002 | -0.891 | -0.478 | -0.395 | -1.066 |
|  | max | 2.233 | 2.581 | 0.813 | 1.003 | 1.018 | 0.551 |
|  | One sample T-Test p value for CFB vs no change | - | - | 0.7614 | - | - | 0.7630 |
| Ln RMS sway [mm] | n | 13 | 13 | 13 | 27 | 27 | 27 |
|  | **mean** | **2.431** | **2.302** | **-0.129** | **1.896** | **1.905** | **0.008** |
|  | SD | 0.618 | 0.619 | 0.386 | 0.510 | 0.494 | 0.340 |
|  | lower 95%CI | 2.058 | 1.928 | -0.362 | 1.694 | 1.709 | -0.126 |
|  | upper 95%CI | 2.805 | 2.676 | 0.104 | 2.098 | 2.100 | 0.143 |
|  | median | 2.531 | 2.284 | -0.089 | 1.941 | 1.892 | 0.088 |
|  | min | 1.560 | 1.581 | -0.816 | 1.081 | 0.621 | -0.720 |
|  | max | 3.640 | 3.898 | 0.533 | 3.564 | 2.843 | 0.596 |
|  | One sample T-Test p value for CFB vs no change | - | - | 0.2498 | - | - | 0.8988 |

Supplementary Table 11: Generalized Anxiety Disorder 7 (GAD-7) and Patient Health Questionnaire Depression Module (PHQ-9) scores in patients with FD at baseline, after 2±1 months, and as change from baseline (CFB). Data are given as number of patients with available data for both collection time points (n), mean, standard deviation (SD), upper and lower 95% confidence interval (CI) of the mean, median, minimum (min), maximum (max), One sample t-Test p value for CFB vs no change as well as number, proportion and 95%CI of the proportion of patients with GAD-7 reduction of ≥4 points or PHQ-9 reduction of ≥5 points.

| **Functional dizziness** | GAD-7 | | | PHQ-9 | | |
| --- | --- | --- | --- | --- | --- | --- |
|  | baseline | 2 months | CFB | baseline | 2 months | CFB |
| n | 38 | 38 | 38 | 39 | 39 | 39 |
| **mean** | **8.4** | **6.3** | **-2.0** | **8.2** | **5.9** | **-2.2** |
| SD | 5.7 | 4.7 | 4.4 | 5.7 | 5.2 | 3.8 |
| lower 95%CI | 6.5 | 4.8 | -3.5 | 6.3 | 4.2 | -3.5 |
| upper 95%CI | 10.2 | 7.9 | -0.6 | 10.0 | 7.6 | -1.0 |
| median | 7.5 | 5.5 | -1 | 6 | 5 | -2 |
| min | 0 | 0 | -16 | 0 | 0 | -15 |
| max | 21 | 21 | 8 | 27 | 25 | 4 |
| One sample T-Test p value for CFB vs no change | - | - | 0.0070 | - | - | <0.001 |
| n patients with reduction  ≥4 (GAD-7) or ≥5 (PHQ-9) | - | - | 11 | - | - | 6 |
| proportion of patients with reduction  ≥4 (GAD-7) or ≥5 (PHQ-9) | - | - | 29% | - | - | 15% |
| upper Clopper-Pearson 95%CI | - | - | 15% | - | - | 6% |
| lower Clopper-Pearson 95%CI | - | - | 46% | - | - | 31% |

Supplementary Table 12: Number of patients (n) and proportion (%) shifted from one GAD-7 category to another after 2±1 months from baseline.

| GAD-7 shift table | | 2 months visit | | | |  |
| --- | --- | --- | --- | --- | --- | --- |
|  |  | minimal anxiety | mild anxiety | moderate anxiety | severe anxiety | total |
| Baseline | minimal anxiety | 11 (29%) | 1 (3%) | 0 (0%) | 0 (0%) | 12 (32%) |
|  | mild anxiety | 3 (8%) | 8 (21%) | 1 (3%) | 0 (0%) | 12 (32%) |
|  | moderate anxiety | 3 (8%) | 1 (3%) | 4 (11%) | 0 (0%) | 8 (21%) |
|  | severe anxiety | 0 (0%) | 3 (8%) | 1 (3%) | 2 (5%) | 6 (16%) |
|  | total | 17 (45%) | 13 (34%) | 6 (16%) | 2 (5%) |  |

Supplementary Table 13: Number of patients (n) and proportion (%) shifted from one PHQ-9 category to another after 2±1 months from baseline.

| PHQ-9 shift table | | 2 months visit | | | |  |
| --- | --- | --- | --- | --- | --- | --- |
|  |  | minimal depression | mild depression | moderate depression | severe depression | total |
| Baseline | minimal depression | 10 (26%) | 1 (3%) | 0 (0%) | 0 (0%) | 11 (28%) |
|  | mild depression | 8 (21%) | 8 (21%) | 1 (3%) | 0 (0%) | 17 (44%) |
|  | moderate depression | 0 (0%) | 2 (5%) | 2 (5%) | 2 (5%) | 6 (15%) |
|  | severe depression | 1 (3%) | 2 (5%) | 1 (3%) | 1 (3%) | 5 (13%) |
|  | total | 19 (49%) | 13 (33%) | 4 (10%) | 3 (8%) |  |

Supplementary Table 14: vHIT and caloric testing results in patients with BVP at baseline, after 2±1 months, and as change from baseline (CFB). Data are given as number of patients with available data for both collection time points (n), mean, standard deviation (SD), upper and lower 95% confidence interval (CI) of the mean, median, minimum (min), maximum (max), One sample t-Test p value for CFB vs no change.

| **Caloric testing** | **vHIT** | | | **Caloric testing** | | |
| --- | --- | --- | --- | --- | --- | --- |
|  | baseline | 2 months | CFB | baseline | 2 months | CFB |
| n | 12 | 12 | 12 | 11 | 11 | 11 |
| **mean** | **0.37** | **0.35** | **-0.02** | **13.2** | **18.6** | **5.5** |
| SD | 0.13 | 0.15 | 0.08 | 20.0 | 18.9 | 14.6 |
| lower 95%CI | 0.28 | 0.26 | -0.06 | -0.3 | 6.0 | -4.3 |
| upper 95%CI | 0.45 | 0.45 | 0.03 | 26.6 | 31.3 | 15.3 |
| median | 0.38 | 0.33 | 0.01 | 5.4 | 12.5 | 1.4 |
| min | 0.11 | 0.13 | -0.18 | 0.7 | 1.4 | -12.5 |
| max | 0.57 | 0.64 | 0.06 | 67.4 | 54.9 | 42.4 |
| One sample T-Test p value for CFB vs no change | - | - | 0.5103 | - | - | 0.2423 |

# Supplementary Figures

**Supplementary Figure 1.** Dizziness handicap inventory (DHI) scores for physical (A, B), emotional (C, D), and functional (E, F) sub-domains in patients with BVP (A, C, E) and FD (B, D, F) at baseline, after 2±1 months, as well as change from baseline (CFB; 2 months - baseline). Each point represents data from one patient. For CFB, means with 95%CI are given.

**Supplementary Figure 2.** Posturography measured in patients with BVP (A) and FD (B) at baseline, after 2±1 months, and as change from baseline (CFB; 2 months - baseline). Each point represents data from one patient. For the CFB, means with 95%CI are given.

**Supplementary Figure 3.** Swaypath (A, B) and RMS sway (C, D) variables from posturographic analysis measured in patients with BVP (A, C) and FD (B, D) at baseline, after 2±1 months, and as change from baseline (CFB; 2 months - baseline). Each point represents data from one patient. For the CFB, means with 95%CI are given.

**Supplementary Figure 4.** vHIT (A) and caloric testing results (B) at baseline, after 2±1 months, as well as change from baseline (CFB; 2±1 months - baseline) in patients with BVP. Each point represents data from one patient. For CFB, means with 95%CI are given.
